# Vaccination against the human cytomegalovirus

**DOI:** 10.1016/j.vaccine.2018.02.089

**Published:** 2019-11-28

**Authors:** Stanley A. Plotkin, Suresh B. Boppana

**Affiliations:** a University of Pennsylvania, Vaxconsult, 4650 Wismer Rd., Doylestown, PA 18902, United States; b UAB School of Medicine, CHB 114, 1600 7th Avenue South, Birmingham, AL 35233, United States

**Keywords:** CMV, Congenital infection, Deafness, Pentamer, Reinfection

## Abstract

The human cytomegalovirus (HCMV) is the most important infectious cause of congenital abnormalities and also of infectious complications of transplantation. The biology of the infection is complex and acquired immunity does not always prevent reinfection. Nevertheless, vaccine development is far advanced, with numerous candidate vaccines being tested, both live and inactivated. This article summarizes the status of the candidate vaccines.

## 1 Introduction

The human cytomegalovirus, here abbreviated as CMV, is perhaps the most ubiquitous of human infections. Although better hygiene and lesser close contact between children and adults have decreased prevalence of CMV in developed countries, virtually 100% of adults in low- and middle-income countries have been infected when young. CMV infects T cells and modifies their responses. It is suspected in contributing to arteriosclerosis and immunosenescence and may promote cancers through an oncomodulatory effect. However, its principal medical importance is as the most common congenital infection throughout the world, causing most commonly hearing loss but also in some cases microcephaly, mental retardation, hepatosplenomegaly and thrombocytopenic purpura. As a generalization, between 1 in 200 and 1 in 30 newborns are infected by CMV transmitted from the mother. The seriousness of the infection in the fetus depends on whether the mother is seropositive or seronegative for CMV. Infections in seronegative pregnant women transmitted to the fetus carry the worst prognosis, but fetuses infected by seropositive mothers may also suffer serious consequences [1].

In addition, CMV is the most common infection complicating transplantation. Solid organ transplant patients who receive a transplant from a seropositive donor may suffer disease and seropositive hematogenous stem cell recipients may reactivate CMV due to immunosuppression [2], [3]. These infections may result in serious disease and rejection of the transplant.

In this article we review efforts to prevent CMV infections and their consequences in susceptible populations through immunization. Such efforts have been pursued for almost 50 years and today there is a wide range of candidate vaccines.

## 2 Congenital CMV infection

Cytomegalovirus is the most frequent cause of congenital infection and an important cause of non-hereditary hearing loss and neurodevelopmental disabilities in U.S. and northern Europe [4], [5]. An accumulation of data over the past decade from resource-limited settings also show that congenital CMV infection is also a significant cause of neurologic morbidity in those populations [6], [7]. However, the awareness of this important fetal infection remains low despite the fact that in the U.S., the number of children who suffer long-term sequelae from congenital CMV approaches the disease burden from well-known childhood conditions such as Down syndrome and fetal alcohol syndrome and far exceeds that caused by other infectious diseases including pediatric HIV/AIDS or invasive *Haemophilus influenzae* type b infection prior to the introduction of vaccination [8]. It is estimated that between 20,000 and 30,000 infants are born each year with congenital CMV infection in the U.S [4], [9]. Approximately 10% to 15% of congenitally infected infants exhibit clinical abnormalities at birth (symptomatic infection) and these findings include petechial and purpuric rash, hepatomegaly, splenomegaly, jaundice with conjugated hyperbilirubinemia, microcephaly, seizures and chorioretinitis [10], [11]. In contrast to the involvement of the central nervous system, the hepatobiliary and hematologic abnormalities resolve spontaneously. The vast majority of infected infants (85–90%) have no detectable clinical abnormalities at birth (asymptomatic infection) and therefore, are not identified early in life [5], [12]. Most infants with clinically apparent or symptomatic congenital CMV infection and approximately 10–15% of those with subclinical or asymptomatic infection develop long-term sequelae [5], [11], [12]. Sensorineural hearing loss (SNHL) is the most common sequela of congenital CMV infection and is seen in about half of symptomatic infants and in about 10–15% of children with asymptomatic congenital CMV infection [11], [13], [9]. Other sequelae seen mostly in children with symptomatic infection include cognitive and motor deficits, vision loss and seizures [11], [8]. The diagnosis of congenital CMV infection is confirmed by demonstrating the presence of infectious virus, viral antigens, or viral DNA in saliva or urine from infected infants [14], [15]. PCR-based assays for the detection of CMV DNA in saliva or urine from neonates are now considered the standard diagnostic methods to confirm congenital CMV infection [16]. The absence of clinical findings at birth coupled with the difficulty in confirming congenital CMV diagnosis retrospectively inasmuch as positive CMV testing of specimens from infants after 3 weeks of age can be the result of postnatally acquired CMV infection are important contributors to the underestimation of the disease burden caused by congenital CMV infection.

## 3 Congenital CMV disease burden in low- and middle-income countries (LMIC)

A number of studies in populations in resource-limited settings over the past decade have demonstrated that congenital CMV infection is an important cause of childhood morbidity [6], [7], [17], [18], [19]. As a higher prevalence of congenital CMV infection is seen in populations with high and often near-universal seroimmunity, a substantial number of infants born in the LMICs are CMV-infected. Based on an estimated 1% birth prevalence of congenital CMV infection, approximately 250,000 and 35,000 infected babies are born annually in India and Brazil, respectively (Table 1). Of those, about 10–15% develop permanent sequelae. The natural history of congenital CMV infection in LMIC has not been well defined except for the data from prospective newborn screening studies in Brazil. These studies demonstrate that the prevalence of CMV-associated hearing loss is similar to that seen in the U.S. and northern Europe [18], [19]. The frequency and severity of other neurodevelopmental sequelae in these populations have not been well characterized.

**Table 1:** Estimated annual live births with congenital CMV infection in various countries.^a^

Country	Birth prevalence of congenital CMV infection	Number of infants born annually with congenital CMV infection
United States	0.5–0.7%	20,000–30,000
Brazil	1.0%	35,000
India	1.0–2.0%	270,000–540,000
Nigeria	1.0–2.0%	65,000–130,000

From references [4], [6]. Birth rates from https://data.worldbank.org/indicator/SP.DYN.CBRT.IN?view=map.

a Estimates of the number of infected infants born annually were derived from annual country-specific birth rate and congenital CMV infection prevalence rates.

## 4 Intrauterine transmission and congenital infection

An important determinant of congenital CMV infection is the prevalence of maternal CMV seropositivity in the population [20]. The prevalence of congenital CMV infection is directly proportional to the maternal seroprevalence such that higher rates of congenital CMV infection are consistently observed in populations with high maternal seroimmunity, which may in part reflect higher exposure (Table 2). This is unlike rubella and toxoplasmosis where primary infection during pregnancy accounts for most vertically transmitted infections [21]. Even within a geographic region, CMV seroimmunity varies among women from different racial, ethnic and socioeconomic backgrounds, translating into distinct epidemiologic patterns of congenital infection. Studies in the U.S. have documented that young maternal age and African American race are independent risk factors for delivering an infant with congenital CMV infection [9], [21]. A recent study from France also reported that young maternal age is a risk factor for having an infant with congenital CMV infection [22].

**Table 2:** Prevalence of congenital CMV infection in low- and middle-income countries.*

Study	Country and time period	Maternal seroprevalence	Newborn screening			Number of newborns with congenital CMV infections (%)		
			Specimens	Laboratory methods	Number tested	Infected		Symptomatic
						N	Prevalence% (95% CI)	
Schopfer, 1978	Ivory Coast	100%	Urine	Culture	2032	28	1.4 (1.0–2.0)	0 (0)
Van der Sande, 2007	The Gambia 2002–05	100%	Urine	PCR	741	40	5.4 (4.0–7.3)	3 (8)
Sohn, 1992	Korea 1989–91	96%	Urine and cord blood	Culture	514	6	1.2 (0.5–2.6)	0 (0)
Tsai, 1996	Taiwan	90%	Urine	Culture, PCR	1000	18	1.8 (1.1–2.8)	2 (11)
Zhang, 2007	China 1997–2000	92–99%	Urine	PCR	1159	71	6.1 (4.9–7.7)	17 (24)
Dar, 2008	India	99%	Saliva	PCR	423	9	2.1 (1.1–4.0)	1 (11)
Luchinger, 1996	Chile 1989–94	98%	Urine, saliva	Culture, PCR	658	12	1.8 (1.0–3.2)	0 (0)
Weirich, 1997	Brazil 1994–95	90%	Saliva	Culture	663	21	3.2 (2.1–4.8)	6 (29)
Yamamot, 2011	Brazil 2003–09	96%	Urine and/or saliva	PCR, culture	12,195	121	1.0 (0.8–1.2)	12 (10)
Noyola, 2003	Mexico 2001	92%	Saliva	Culture	560	5	0.9 (0.4–2.1)	0 (0)
Estripeaut, 2007	Panama 2003–04	84%	Urine	PCR	317	2	0.6 (0.2–2.5)	1 (50)
Dar, 2017	India 2010–12	99%	Saliva	PCR	1720	20	1.2 (0.72–1.8)	1 (5)
Manicklal, 2014*	South Africa 2012	Unk	Saliva	PCR	748	22	2.9 (1.9–4.4)	ND
Olusanya, 2015	Nigeria 2012–13	Unk	Saliva	PCR	263	10	3.8 (2.1–6.9)	ND

Adapted from Lanzieri et al. [6].

* Study was conducted in HIV-exposed infants.

Although it has been known since the 1980s that congenital CMV infection can occur in children born to mothers who are CMV-infected prior to pregnancy (non-primary infection), the relative contributions of primary and non-primary maternal infection to congenital CMV infection and CMV-associated hearing loss and other neurologic sequelae have been recognized only recently. A systematic review and modeling of the data in the U.S. suggested that about two-thirds to three-quarters of all congenital CMV infections occur in children born to women with non-primary maternal infection [23], [24]. However, data from prospective studies in the U.S. to confirm these predictions are not available. Thus, it can be assumed that at least half of congenitally infected infants in high income countries are born to women with preexisting seroimmunity and in populations with high seroprevalence such as low-income minority women in the U.S. and the vast majority of women in LMIC, most infected infants are born to women with non-primary CMV infection.

A recent large newborn CMV screening study at two different maternity units in Paris, France where the overall maternal seroprevalence was 61% showed that about half of all CMV-infected babies were born to women with non-primary CMV infection during pregnancy [22]. The study also reported that a similar proportion of infected infants were symptomatic in both primary and non-primary groups. In a subset of 2378 women at one of the maternity units, prenatal sera collected between 11 and 14 weeks gestation were tested for CMV antibodies. The rate of congenital infection was fourfold higher in infants born to originally seronegative mothers compared to women seropositive before pregnancy. A retrospective study of mothers with non-primary infection during pregnancy in Italy showed that 3.4% (7/205) of newborns were infected in utero [25].

Studies in France [22], Italy [25] the United States [26], [27] and Brazil (unpublished) have shown that intrauterine transmission of CMV is considerably lower in women with preexisting immunity compared to those with primary infection during pregnancy. This conclusion is derived from comparisons of the rates of fetal infection after primary infection with the rates of fetal infection from previously seropositive women. Thus, maternal immunity provides considerable protection against transmission to the fetus. Thus, maternal immunity is partly protective against fetal infection.

The risk factors for acquiring CMV during pregnancy in seronegative women include increased exposures to CMV such as direct care of young children, sexually transmitted infections and other indices of sexual activity [21]. Although the risk factors for non-primary maternal CMV infection have not been defined, it is likely that similar to primary maternal infection, increased exposure to other individuals excreting CMV is associated with non-primary infection. While the mechanisms have not been defined, reactivation of endogenous virus or reinfection with a new virus strain have been suggested as possible virus sources leading to intrauterine transmission of CMV in non-primary infections [5], [20]. Recent studies have demonstrated that exposure to a new strain of virus can lead to reinfection of seropositive women, intrauterine transmission and congenital infection [28], [29]. The characteristics of antiviral immune responses that provide protection against intrauterine transmission are also not well understood. In women with primary CMV infection during pregnancy, lower neutralizing antibody levels, slow development of antibody to the viral pentamer proteins, and a slow increase in IgG avidity have been associated with fetal infection [30], [31], [32]. A lag in the development of CMV-specific CD4^+^ and CD8^+^ T-cell responses was also observed in women with primary maternal CMV infection who transmitted virus to their infants. However, no differences in the duration of viremia and the peak viral load were observed between women with and without intrauterine transmission.

## 5 Transplant disease

CMV infection is the most common infectious complication of transplantation, both solid organ and hematogenous stem cell. In the case of solid organ transplantation, including kidney, liver, lung and other, the most dangerous situation is when a CMV seronegative recipient receives an organ from a CMV seropositive donor [33], [34], [35], [36]. In that situation CMV infection is almost certain, and disease is common. In the case of kidney transplants, without antiviral prophylaxis, about one third of seronegative recipients of a kidney from a seropositive donor will have CMV disease. Interestingly, even seropositive recipients may have CMV disease when transplanted with an organ from a seropositive donor. Inasmuch as seropositive recipients who receive an organ from a seronegative donor have much less CMV disease, it suggests that the problem for seropositive recipients is superinfection with a new strain under the influence of immunosuppression, rather than reactivation.

The situation after hematogenous stem cell transplant (HSCT) is different. In that case it is reactivation under the influence of immunosuppression that seems to be the danger. Inasmuch as latency of CMV occurs not only in circulating T cells but also in lymph nodes, endothelial cells, macrophages and other sites, it is not surprising that reactivation is a problem [37], [38], [39].

Antiviral prophylaxis and/or treatment are practiced routinely in transplant centers to prevent serious CMV disease and has had considerable but not complete success. As discussed in the section on vaccines below, vaccination has had some early success in reducing the severity of CMV disease and definitive trials are underway to determine if such approaches are sufficiently efficacious.

## 6 CMV vaccines

It appears that antibodies are necessary to prevent acquisition and spread of CMV by seronegatives, but T cells responses are needed to suppress reactivation of the virus in seropositives. The development of CMV vaccines began in the 1970s soon after the toll of the virus on infants in utero and transplant recipients became obvious. Two vaccine strains were attenuated starting with viruses that had been isolated for laboratory work: AD-169 and Towne [40], [41]. The AD169 attenuated strain was soon abandoned, but the Towne attenuated strain went on to extensive testing in solid organ transplant recipients and normal male and female volunteers [42]. Recipients of kidney transplants who were administered the Towne attenuated strain virus were shown to be highly protected against serious CMV disease and rejection of the graft. Protection against infection, however, was not statistically significant. The investigational Towne strain vaccine could protect humans against a challenge with unattenuated CMV, but naturally acquired immunity protected against a higher dose challenge than did the vaccine [43]. Also, the attenuated strain failed to prevent natural acquisition of CMV by women exposed to children in day care [44]. The reason for the latter failure is unknown.

The next important development was the purification of a surface protein of CMV called glycoprotein B, or gB, because of homology with a glycoprotein of other herpesviruses. When combined with the MF59 oil-in-water adjuvant, good levels of neutralizing antibodies were produced in humans after three injections over a six-month period [45], [46]. This regimen was tested twice in comparison with placebo in young women naturally exposed to CMV, and in both cases there was moderate reduction in acquisition, but antibodies and efficacy faded quickly. A booster injection did restore antibody levels. In addition, when the subunit gB protein was combined with the AS01 adjuvant that stimulates toll-like receptor (TLR) 4, higher and more prolonged levels of anti-gB antibodies were elicited in humans, but that adjuvanted vaccine was never tested for efficacy. Significantly, the investigational subunit gB vaccine gave remarkable protection against CMV disease in solid organ transplant patients, suggesting the importance of antibodies in that situation [47]. The fact that gB is a trimeric fusion protein suggests the possibility that a more immunogenic prefusion form may exist, but this has not yet been demonstrated.

In the year 2000 an important event took place: the publication of a vaccine priority document by the Institute of Medicine (now the National Academy of Medicine) of the United States [48]. CMV was placed in its highest priority for vaccine development. This event strongly stimulated vaccine manufacturers and biotechnology companies to work in this field.

Another event that has proved important is the discovery by researchers at Princeton University that a pentameric complex of proteins was present on the surface of CMV and that this structure, consisting of glycoprotein H (gH), glycoprotein L (gL), and the products of genes UL128, 130 and 131, elicited far more neutralizing antibodies than gB [49].

Parallel work done by a team at the University of Pavia in Italy showed that in pregnant women infected by CMV a rapid response to the pentameric complex was associated with protection against transmission to the fetus [50]. This discovery has since driven much of the vaccine field.

Table 3 lists both live and inactivated candidate vaccines against CMV. An attempt was made to increase the immunogenicity of the Towne attenuated virus by making recombinants with the Toledo low passage “wild” CMV. Four recombinants were tested in small numbers of humans and one turned out to be suitably immunogenic [44]. However, another attractive approach that in principle combines safety with immunogenicity is a replication-defective virus. This candidate is made in cell culture using a CMV with two proteins rendered potentially unstable by chemical combination but stabilized by a chemical called Shld 1. On injection into humans in the absence of Shld 1, the virus cannot form infectious particles but does express immunogenic proteins. In phase 1 trials the replication-defective virus gave good immune responses [51].

**Table 3:** CMV vaccines in development.

Type of vaccine	Developer	Ref. #
Attenuated strain (Towne)	Wistar Inst./Med Coll VA	[41]
Recombinants with wild virus (Towne-Toledo)	Medimmune	[44]
Replication-defective virus	Merck	[51]
Vectored:		
Canary Pox	Sanofi	[52]
MVA	City of Hope	[57]
Adeno	Queensland Inst.	[58]
LCMV	Hookipa	[55]
VSV	Yale	[59]
Recombinant gB glycoprotein with adjuvant	Sanofi Pasteur, GSK	[45], [46], [47]
Soluble Pentamers	Redbiotech, GSK, Humabs	[49]
DNA plasmids	Astellas, Inovio	[61], [63]
Self-replicating RNA	Moderna	[54], [62]
Peptides	City of Hope	[64]
Dense bodies	Vaccine Project Management (Germany) and Serum Inst. India	[61]
Virus-like particles	Variations Bio	[56]

A number of vaccine candidates are based on vectored genes of CMV, in particular gB and the tegument phosphoprotein 65 (pp65). Immunogenicity has been demonstrated, and safety in some cases. In principle they should be protective in transplant patients and perhaps in seronegative normal subjects [52], [53], [54], [55], [56], [57], [58], [59].

Inactivated candidate vaccines are also listed in Table 3. Aside from the investigational gB subunit vaccines mentioned above, peptides, DNA and mRNA vaccines are significant candidates [60], [61], [62]. DNA plasmids coding for pp65 and gB have shown preliminary evidence of efficacy in transplant recipients [63]. In addition, a virus-like particle with gB on the surface has shown surprisingly high induction of neutralizing antibodies in animals, pp65-derived peptides combined with a tetanus toxin epitope have been immunogenic in man, and so-called dense bodies harvested from cell cultures of CMV contain all of the viral antigens [64], [65].

Thus, there is no lack of candidate CMV vaccines for use in humans. For prevention of infections in all forms of transplantation, induction of both antibody and T-cell responses are essential. At this stage of our knowledge it appears that both gB and pentamer should be included in vaccines designed to prevent fetal CMV infection and/or disease.

## 7 How a CMV vaccine would be used

There are several unanswered questions about the feasibility of a CMV vaccine, but there are also some clear answers. CMV is acquired by contact with saliva, sexual secretions and transplantation. In principle, the populations that could benefit from protection against CMV are four: seronegative women of child-bearing age, seropositive women of child-bearing age, recipients of solid organs (SO) donated by CMV seropositive individuals, and seropositive hematogenous stem cell (HSC) recipients. The case for the two transplant populations is most evident: morbidity from CMV is considerable, antiviral prophylaxis is expensive, not completely effective, and cannot be continued indefinitely. Ideally, a CMV vaccine would be given before transplantation, but for HSC trial patients who acquire a new immune system, vaccination should continue after transplant. Although not 100% certain, it appears that CMV antibodies are needed by SO trial recipients [47], while HSCT recipients need reinforcement of T-cell immunity against CMV [63]. The state of vaccine development arguably is such that definitive evidence for efficacy of these two approaches could be obtained in 1–3 years.

The situation for women of child-bearing age is less clear. However, the multiplicity of candidate vaccines discussed above argues that we are in the position of being able to induce neutralizing antibodies against gB and pentamer as well as CD4^+^ and CD8^+^ T-cell responses against those two surface antigens, plus the pp65 matrix protein. Evidence for the importance of immune responses to these antigens in prevention of acquisition (gB) and transmission to the fetus (pentamer) has been acquired, and although controversial, passive antibodies may protect the fetus [66], [67]. Thus, CMV vaccination in North America, Europe and elsewhere where many women approach pregnancy without antibodies to CMV is justified. In addition, modeling suggests that vaccination of toddlers, similar to the practice for rubella vaccine, would offer strong indirect protection to women, many of whom are infected by their first child during a subsequent pregnancy [68]. If duration of vaccine-induced protection is long enough, CMV vaccination could be offered to pre-adolescents at the same time as HPV vaccine.

At this point, uncertainty exists about the immunological deficits that allow reinfection of seropositive individuals, including pregnant women, and studies to define those deficits are urgently needed. Although the incidence of abnormalities in infants born to reinfected mothers is less than those born to mothers who had primary infection, reinfection can cause serious consequences [23], [24]. Is reinfection the result of low pentamer antibodies, low T-cell responses, or a high force of infection? Answers to these questions are badly needed, as the vast majority of women in the world who live in LMICs have been infected with CMV in childhood and are seropositive. Nevertheless, there is a burden of fetal and newborn disease that should be prevented by vaccination, as contact between asymptomatically infected children and mothers cannot be eliminated.
